# Incisión de clamshell con esternotomía media como abordaje del aneurisma de arco aórtico complicado

**DOI:** 10.47487/apcyccv.v3i3.225

**Published:** 2022-09-30

**Authors:** Gerber Polo-Gutiérrez, Luis Felipe Bustinza-Carassa, Yemmy Pérez-Valverde, Yuler Abono Sánchez

**Affiliations:** 1 Instituto Nacional Cardiovascular INCOR. Lima, Perú. Instituto Nacional Cardiovascular INCOR Lima Perú

**Keywords:** Aneurisma de la Aorta, Rotura de la Aorta, Pectus Excavatum, Síndrome de Marfan, Aortic Aneurysm, Aortic Rupture, Pectus Excavatum, Marfan Syndrome

## Abstract

El tratamiento del aneurisma de arco aórtico constituye uno de los mayores desafíos en la cirugía de aorta. Presentamos el caso de una mujer joven con antecedente de síndrome de Marfan, Pectus Excavatum severo y cirugía de Bentall, que ingresó a cirugía de emergencia por aneurisma de arco aórtico roto-contenido. Se logró un abordaje exitoso mediante una incisión de *Clamshell* asociada a reesternotomía media.

## Abstract (en)

Surgical treatment of aortic arch aneurysm is one of the greatest challenges of aortic surgery. We present a young woman with Marfan syndrome, severe Excavated Pectus and previous Bentall procedure, who underwent emergency surgery for ruptured aortic arch aneurysm. We achieved a successful approach through a Clamshell incision associated with a median re-sternotomy.

## INTRODUCCIÓN

El síndrome de Marfan (SM) es una enfermedad hereditaria autosómica dominante y multisistémica. Las principales manifestaciones cardiovasculares del SM son la dilatación y/o disección de la raíz aórtica, de la aorta ascendente proximal y de la aorta distal. La afectación al sistema esquelético puede originar anomalías torácicas como el *Pectus Excavatum* (PE), en un 0,5-5,3% ^1^.

La cirugía abierta es el abordaje ideal para enfermedades de la aorta torácica en los pacientes con SM, obteniéndose una tasa de isquemia de la médula espinal que varía del 4 al 7% y la mortalidad del 5 al 14% ^2^. Después de una cirugía de raíz aórtica en pacientes con SM se pueden desarrollar pseudoaneurismas de los botones coronarios, aneurismas o disección de la aorta nativa remanente, incluido el arco o cayado aórtico (CA), y así requerir reintervenciones ^3^^,^^4^. El principal factor de riesgo para la necesidad de reintervención en el CA y la aorta distal después de una disección tipo A reparada es una luz falsa permeable, especialmente si el desgarro de entrada primario no se resecó durante la cirugía inicial ^5^.

Presentamos el caso de una paciente joven con SM, PE, con antecedente de cirugía cardiaca y un aneurisma gigante del CA.

## REPORTE DEL CASO

**Figura 1:**
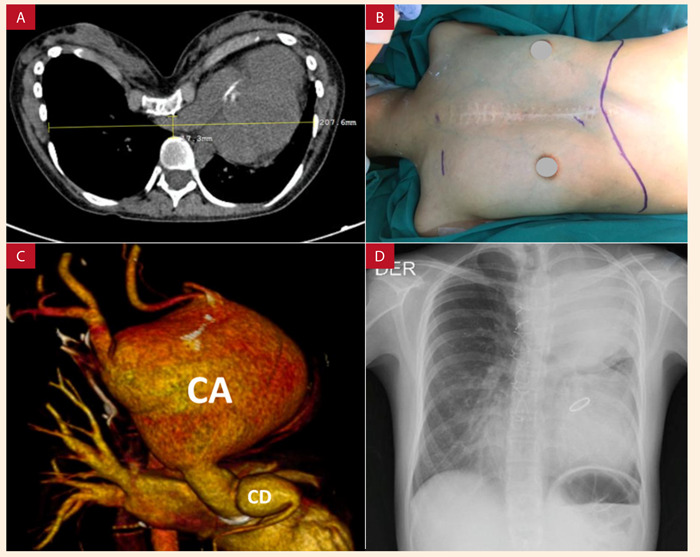
A) Tomografía de tórax - corte axial, donde se evidencia un índice de Haller de 12, correspondiente a un PE Severo. B) Se observa el PE severo, la cicatriz de la esternotomía previa y el marcaje por donde se realizó la incisión de *Clamshell*. C) Reconstrucción por angiotomografía preoperatoria donde se observa la severa dilatación aneurismática fusiforme de 115 mm x 80 mm x 100 mm, localizado en todo el cayado aórtico (CA) y en los primeros diez centímetros de la aorta torácica descendente; asimismo, se observa la dilatación en la coronaria derecha (CD) proximal. D) Radiografía de tórax posterior-anterior con radioopacidad de aproximadamente el 50% del hemitórax superior izquierdo, correspondiente con el aneurisma del cayado aórtico.

Mujer de 21 años con SM y PE severo (índice de Haller de 12) (Figura 1A, 1B), con antecedente de cirugía de Bentall mecánico más ligadura de *Ductus Arterioso* persistente a los 12 años. Desde seis meses antes del ingreso presentaba dolor torácico a moderados esfuerzos, disnea clase funcional II y disfonía por lo que fue referida a nuestro centro para estudio. En la tomografía con contraste encontramos un aneurisma del arco aórtico de 115 x 80 x 100 mm de diámetro que comprometía también los diez primeros centímetros de la aorta descendente, además de un aneurisma de la arteria coronaria derecha (CD) en su porción proximal (Figura 1C, 1D) Durante su hospitalización cursó con dolor torácico intenso por lo que fue programada para cirugía de emergencia.

**Figura 2:**
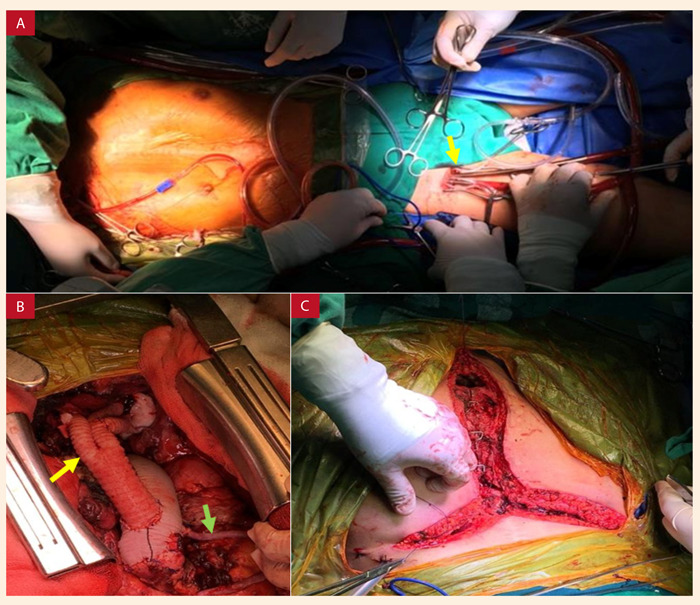
A: Fotografía intraoperatoria que muestra la canulación periférica a través de los vasos femorales (flecha); asimismo, la exposición de la arteria axilar derecha para la perfusión cerebral selectiva anterógrada. B) Se muestra el reemplazo del arco aórtico con injerto de dacrón y el *debranching* a vasos supraaórticos (flecha amarilla), también el baipás a coronaria derecha con vena safena (flecha verde). C) Cierre de la herida operatoria, se muestra el abordaje quirúrgico asociado: incisión en *Clamshell* más esternotomía media.

En la cirugía se realizó canulación arterial y venosa femoral (Figura 2A). Se realizó perfusión cerebral por ambas arterias carótidas, por lo que se anastomosó un injerto de dacrón de 8 mm en la arteria axilar derecha y se disecó la arteria carótida izquierda. Se realizó una incisión de *Clamshell* en el quinto espacio intercostal y se liberó las adherencias parcialmente, luego del ingreso a circulación extracorpórea (CEC) se complementó el abordaje con reesternotomía media y se completó la liberación de adherencias. Se llevó a arresto circulatorio con hipotermia moderada y perfusión cerebral anterógrada; se abordó el aneurisma de CA, se retiró el tejido aneurismático, se reemplazó el CA con injerto de dacrón N.º 26, realizándose en primer lugar la anastomosis distal en aorta torácica descendente proximal excluyendo el aneurisma y luego el *debranching* a tronco braquiocefálico y a carótida común izquierda (Figura 2B). Después de 60 min se finalizó el arresto circulatorio y la perfusión cerebral selectiva y se reingresó a CEC; posteriormente, se realizó *debranching* a arteria subclavia izquierda.

Debido a que el segmento proximal de la coronaria derecha (CD) estaba aneurismática, se realizó exéresis de ese tejido, se creó un neoseno con pericardio bovino y se colocó un baipás a CD con vena safena; finalmente, el injerto de dacrón se anastomosó a la aorta ascendente. Tras la salida de CEC, debido al sangrado excesivo, se realizó un empaquetamiento mediastinal con gasas. Durante el posoperatorio inmediato (2 h) la paciente reingresó a sala de operaciones por presentar sangrado excesivo debido a un desgarro de la vena cava superior, luego de revisar la hemostasia se retiraron las gasas que se habían dejado en la primera cirugía y se cerró la herida por planos (Figura 2C). La estancia hospitalaria total fue de 30 días. 

**Figura 3:**
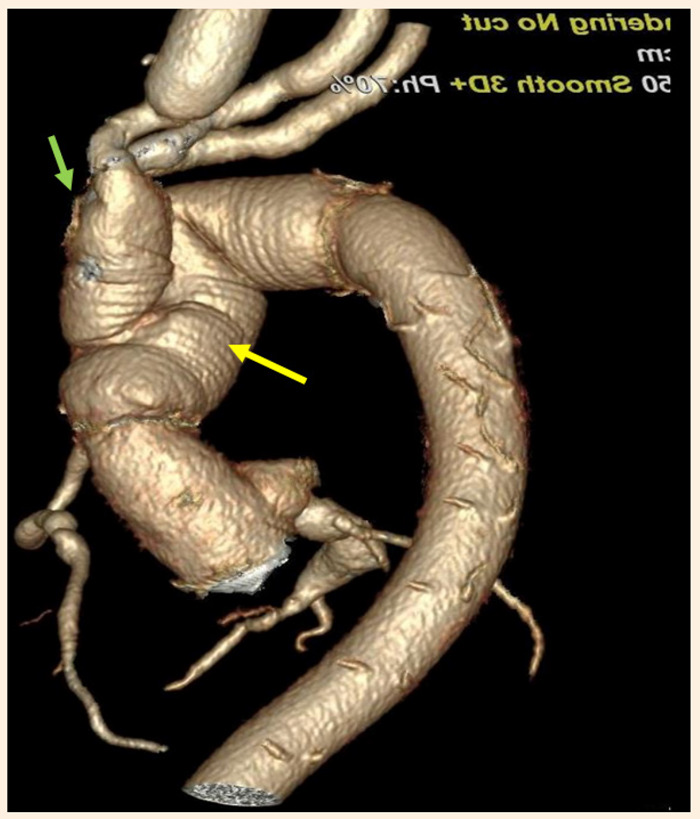
Reconstrucción por angiotomografía posoperatoria que muestra que el arco aórtico y la aorta descendente proximal se reemplazaron con el injerto protésico (flecha amarilla), también se aprecia el debranching a los vasos supraaórticos (flecha verde).

A los 7 meses de seguimiento, la paciente se ha mantenido con un estado cardiovascular y neurológico estables, con control tomográfico donde se evidenció la permeabilidad de los injertos de cayado - aorta descendente proximal, de los injertos a vasos supraaórticos, así como del puente venoso a CD y de la vena cava superior reparada (Figura 3).

## DISCUSIÓN

El aneurisma de la aorta torácica es una causa importante de morbilidad y mortalidad ^2^. La sintomatología es variable y depende del tipo de aneurisma, de su localización y relación con las estructuras vecinas. Nuestra paciente presentó disnea y disfonía debido a esta patología ^6^.

La angiotomografía de la aorta y de los vasos periféricos en el prequirúrgico es vital para decidir el momento quirúrgico y realizar la planificación operatoria. Un diámetro superior a 50 mm en la angiotomografía, aumenta el riesgo de rotura y disección hasta en un 45% ^7^. En nuestro caso el diámetro se encontraba más de dos veces de este valor de riesgo, complicándose con una ruptura contenida, por lo que fue intervenida de emergencia.

La paciente tenía como antecedente una cirugía de Bentall por esternotomía media. La reentrada al mediastino en casos como estos es un punto crítico debido a que las adherencias del ventrículo derecho, del injerto de aorta y/o de la vena innominada, hacen susceptible de ocasionar lesiones al corazón o a los grandes vasos. La complicación adicional de ruptura contenida de la aorta de la paciente hizo necesario el ingresar a CEC por vía femoral y llevar a hipotermia sistémica antes de la reesternotomía ^8^.

La complejidad del abordaje quirúrgico fue mayor, porque la joven paciente presentaba PE. La cirugía cardiaca, y especialmente la del cayado aórtico en aquellos pacientes con PE, es un gran desafío, debido a la desviación y desplazamiento posterior del esternón y la lateralización izquierda del corazón y de los grandes vasos. La esternotomía media convencional no permite trabajar de forma fácil y segura sobre el arco distal, más allá del origen de la arteria subclavia izquierda y sobre la aorta descendente ^9^. La toracoesternotomía bilateral o incisión en «concha de almeja» o *Clamshell*, que consiste en una toracotomía anterior bilateral con esternotomía transversa, permite una buena exposición de las estructuras del mediastino, como los nervios frénico y laríngeo recurrente, reduciendo así la posibilidad de lesión ^10^.

Esta incisión es utilizada para el reemplazo total del CA, incluido el CA distal y algún segmento de la aorta torácica descendente. Se reporta la preferencia por la población femenina, joven (mediana de edad, 42), y en pacientes con cirugía cardíaca previa ^10^. Dentro de las complicaciones informadas se encuentran: la infección de herida con reintervención (12 %), además de una baja de reintervención por hemorragia y de complicaciones pulmonares ^10^^,^^11^. Con la incisión de *Clamshell* la supervivencia a 1 año es del 94% ^10^. Se puede asociar este abordaje con una esternotomía media para los casos que requieren una exposición más amplia. Todas esas características mencionadas sumaron para adoptar esta estrategia quirúrgica en el caso reportado.

La intervención sobre los vasos supraaórticos, cuando se reemplaza el CA, conlleva el riesgo de lesión neurológica permanente, siendo la complicación más común y la principal causa de muerte de esta intervención ^12^. Una adecuada neuroprotección durante este tipo de cirugías es esencial. Las estrategias para la neuroprotección siguen siendo un área en investigación; sin embargo, se ha reportado que la hipotermia moderada y la perfusión cerebral anterógrada son estrategias seguras y efectivas ^12^^)^ en nuestra paciente, a los 7 meses de seguimiento, no se evidenció ninguna lesión neurológica.

En conclusión, la incisión de *Clamshell* asociada a esternotomía media, en conjunto con hipotermia moderada y perfusión cerebral anterógrada para la neuroprotección, constituyeron una adecuada estrategia en la cirugía de CA en esta paciente con cirugía cardiaca previa, SM y PE. 
